# About Face: Evaluating and Managing Tactile Impairment at the Time of Autism Diagnosis

**DOI:** 10.1155/2015/612507

**Published:** 2015-10-28

**Authors:** Louisa M. T. Silva, Mark Schalock, Kristen R. Gabrielsen

**Affiliations:** ^1^Teaching Research Institute, Western Oregon University, Post Office Box 92, McMinnville, OR 97128, USA; ^2^Teaching Research Institute, Western Oregon University, 345 N. Monmouth Avenue, Monmouth, OR 97361, USA

## Abstract

Evaluation for sensory impairment is a routine part of autism diagnosis. Sensory impairment of hearing, vision, or touch results in developmental delay and must be addressed before delay can resolve. Recent studies confirm that tactile impairment is present in autism and can be effectively treated with a tactile stimulation protocol. The research suggests a change in management at the time of autism diagnosis to include evaluation and treatment of tactile impairment. Here we validate screening and management tool for tactile impairment, the Autism Touch and Self-Regulation Checklist, in 404 typical and autistic preschool children. The tool assesses tactile impairment by location and severity. Autistic children were distinguished by mixed pain and numbness on multiple areas including the face and mouth (*F* = 412.1 (1,402);*p* < .000). Oral-facial tactile impairment interferes with the tactile stimulus to orienting. We hypothesized that oral-facial tactile impairment and difficulty orienting are predictive of ASD and that severity of tactile impairment is predictive of severity of ASD. Questions evaluating oral-facial and orienting responses correctly predicted 91% of the autism group. Severity of tactile impairment correctly predicted 81% of mild versus severe ASD. Results underscore the importance of evaluating and treating tactile impairment at the time of autism diagnosis.

## 1. Introduction

The evaluation for sensory impairment is a routine part of the autism diagnosis. Sensory impairment of hearing, vision, or touch results in developmental delay and must be treated before normal development can resume. The inclusion of sensory problems in the diagnosis of autism in 2013 [1] leads to the recognition that although tactile abnormalities are universal and markedly severe in children with autism, the sense of touch has not been fully evaluated and cannot be presumed to be intact [2–4].

Recent studies confirm that tactile impairment is universally present in children with autism and describe a distinguishing clinical presentation characterized by mixed pain and numbness on multiple areas of skin including the face, mouth, hands, and feet [5, 6]. Tactile impairment is severe compared with other developmental disabilities and associated with delay of early self-regulation milestones [5]. The presentation is typical of early-onset sensory neuropathy [7].

A number of studies report that tactile impairment in children with autism can be reduced with a tactile stimulation protocol stimulating circulation to small sensory nerves in the skin: normalization of tactile symptoms by 50% is reported following five months of daily treatment [8–11]. Treatment also normalized autistic behavior by one-third and increased social/language development. In 2015, a large study replicated the results and confirmed that treatment was effective in high- and low-functioning children [12]. This was a breakthrough in treatment for autism.

The research suggests a change in management at the time of autism diagnosis to include evaluation and treatment of tactile impairment. To this end, clinicians require screening and management tool for tactile abnormalities that identifies signs of tactile impairment by severity and location and measures response to treatment. Only one such management tool is currently available.

The Autism Touch and Self-Regulation Checklist (ATSC) is a 59-item tool for managing tactile impairment in children with autism (see http://qsti.org/ATSC.pdf). The checklist assesses tactile symptoms by severity and location and seeks confirmation of severity of tactile impairment by simultaneously assessing effects on touch-dependent, early self-regulation milestones (e.g., orientation-attention, self-soothing, and sleep) [6]. Since its being developed as a research tool, the ATSC has been refined for use by clinicians and is available free of charge at http://qsti.org/ATSC.pdf.

In this paper, we present updated validation data for the ATSC and investigate two hypotheses. The first is that the presence of tactile impairment on the face and mouth is predictive of ASD. We note that tactile impairment of the face and mouth would disrupt tactile stimulation of the orienting reflex and reduce face-to-face orienting and social attention on that basis [13]. The second is that severity of tactile impairment is predictive of severity of autism. We analyze the ASD data pool to determine whether we can distinguish between severely and mildly affected children on that basis.

The research questions are as follows:

1. Is the Autism Touch and Self-Regulation Checklist a valid instrument for screening for and managing signs of tactile impairment in autism?
2. To what extent do location of tactile signs on the face and mouth and failure to orient correctly identify the children with autism in the group?
3. To what extent does severity of tactile impairment distinguish between children with severe autism and those with mild autism?

## 2. Materials and Methods

### 2.1. Participants

Data from 404 children aged 24 to 72 months were collected for this study: 199 children with Autism Spectrum Disorder (ASD) and 205 typically developing children. All informants were primary caregivers for the children concerned.  Table 1 provides demographic information for each group.

### 2.2. Measures

Initially the ATSC was organized into individual sensory and self-regulatory domains. The choice of self-regulatory questions reflects disturbances commonly reported by parents in the main self-regulatory milestones in the first 3 years of life: orientation-attention, self-soothing, sleep, digestion, toilet training, and emotional and behavioral self-regulation.

The individual items were developed and selected through an iterative process by conducting a review of more than 100 interviews of parents with young autistic children in which parents were asked open-ended questions about their child's sensory and self-regulatory responses to ordinary, daily-life situations. As it became evident from parent interviews that sensory responses to both injurious and noninjurious stimuli were grossly abnormal, we created six sensory subdomains: touch-pain, auditory, visual, taste-smell, hyperreactive to noninjurious stimuli, and hyporeactive to injurious stimuli. The hyperreactive and hyporeactive subdomains do not have their own separate item. The hyperreactive subdomain was drawn from all sensory subdomains, and the hyporeactive subdomain is drawn from the oral-tactile subdomain only.

We created an additional category for analysis when we realized that parents were reporting abnormal touch-pain responses in multiple areas of the body, including the face, head, fingers, toes, skin, and diaper area. The ATSC labels touch-pain items by their respective body area; for example, “difficulty with haircuts” is assigned to the head and “difficulty cutting fingernails” is assigned to the fingers. Information regarding the number of areas involved was extracted from the sensory domain and analyzed separately.

Over an initial five-year period, we submitted the ATSC to an ongoing development and refinement process in which items were evaluated for inclusion, exclusion, and clarification of terminology. The ATSC caregiver report is suitable for use by caregivers who have an elementary school education and is available in English, Spanish, Portuguese, and Chinese. The results of this initial validation effort were published previously [6].

The ATSC has been refined since its initial design eight years ago in three ways. First, items in the oral domain reflecting responses to texture and pressure in the mouth have been moved, and a single oral-tactile domain was created. Second, a toilet training domain was added to assess self-regulation of toilet training, and several items that failed to differentiate between ASD and typical and other developmental delays groups have been deleted. Finally, the original checklist contained 56 items across 10 sensory and self-regulation domains. The revised checklist contains 59 items assessing nine domains.

Table 2 lists all ATSC items according to our revised organization by oral-tactile and self-regulatory domain and identifies the additional items related to the sensory domains of vision, hearing, and smell that can be used for clinical purposes if needed. Items are rated* never* (0),* rarely* (1),* sometimes* (2), or* often* (3). Domain scores are obtained by summing the individual items. Table 2 also shows the prevalence of responses scored* sometimes* or* often* across the three groups of children.

### 2.3. Data Collection

We collected ATSC data on children with autism from several research projects completed over the past seven years with Western Oregon University Institutional Review Board approval. Inclusion criteria for the children with autism were as follows: age less than six years, receiving state-sponsored early childhood special education services for autism, absence of other severe disabilities such as cerebral palsy, and taking no psychotropic medication. Children* with a preexisting autism diagnosis from an autism evaluation center or developmental pediatrician* were recruited from six regional autism programs across Oregon. The diagnostic evaluations were reviewed and autism diagnosis was confirmed by DSM-IV criteria prior to final acceptance into the study [14]. All data from these groups were collected prior to random assignment into treatment and control groups associated with specific studies conducted by the authors from 2008 to 2014.

We recruited parents of typically developing children to complete the surveys from one child care center, three mother support groups, and one toddler drop-in play center in Oregon. Parents completed the surveys on convenience basis. Inclusionary criteria for the children included the following: ages 3–6; no educational or medical diagnosis of autism; absence of developmental delays (DD) or previous assessment for DD; and absence of chronic medical conditions.

While ages of children in the typically developing and autism groups are similar, gender distribution is highly unequal. Given that the prevalence of Autism Spectrum Disorder is much higher in boys than girls, analyses were conducted to assess whether boys and girls in the typically developing group differed on the ATSC. To determine whether this influences the results, two sets of analyses were conducted. First, analyses were conducted to determine whether there are differences on measures by gender in the typically developing group.

Chi-square analyses were conducted to determine whether the prevalence of abnormal sensory responses differed by boys and girls on oral-tactile, vision, hearing, smell domains and the number of senses involved. Chi-square values ranged from .024 on hearing to 2.47 on oral-tactile domain. Associated *p* values ranged from .877 to .116. Consequently, there does not appear to be a significant difference between boys and girls on the prevalence of sensory issues.

Additionally, ANOVA/MANOVA was conducted comparing boys and girls on the outcome measures presented in Table 5. No significant differences were found. *F* values ranged from .002 for face-mouth to 2.00 for constipation. Associated *p* values ranged from .960 to .158.

To further test whether the unequal distribution was problematic, the analyses in Table 5 and the ROC curve analyses were replicated comparing only boys from the typically developing and ASD groups. *F* values ranged from 404.1 for face-mouth and orientation and attention to 23.3 for constipation. All *F* values were significant beyond the .000 level. AUC values decrease very little (usually by .001) and sensitivity and specificity scores typically increase by less than .025.

While the distribution in gender was unequal between the typically developing and Autism Spectrum Disorder groups, this did not overly influence the results presented here.

### 2.4. Data Analysis

To determine the prevalence of oral-tactile and self-regulatory symptoms in children with autism and typical development, simple frequency analyses were conducted on each item. The percentage of responses of* sometimes* or* often* was computed for each group.

We also investigated the ATSC's ability to discriminate between children with ASD and those typically developing on the basis of the number of senses involved and the number of areas of body involvement by analyzing frequency distributions using *χ* ^2^ analyses and the median test. To determine the prevalence of abnormal sensory responses and self-regulatory delay, we set the criterion as the mean score for typically developing children plus 1 standard deviation [15]. We conducted a parallel analysis on areas of the body affected.

To determine reliability, we calculated internal consistency estimates (Cronbach's alpha) for the total ATSC score, the sensory and self-regulation domain, and the individual subdomains for each population. We also calculated test-retest stability on a subsample of children in the control condition over a four-month time interval.

The ATSC's ability to differentiate between children with autism and typically developing children was evaluated with multivariate analysis of variance (MANOVA). In addition, response operator characteristic (ROC) curve analysis was conducted to determine the overall adequacy of the ATSC and various domains and subdomain composite scores as a screening tool for autism and levels of sensitivity and specificity at various cut-off scores.

The above set of analyses was replicated to determine whether the ATSC could effectively differentiate between severe and mild levels of severity of autism, as determined by a composite of measures. The ROC curve analyses were conducted to determine the overall adequacy of the ATSC and various domains and subdomain composite scores as a screening tool for severe versus mild autism and levels of sensitivity and specificity at various cut-off scores.

To create a composite severity of autism score we combined scores from the Pervasive Developmental Disorders Behavior Inventory Parent Version [16], the Autism Behavior Checklist [17], the Vineland Adaptive Behavior Scales [18], the Childhood Autism Rating Scale [19], and the Preschool Language Scale 5th Edition [20]. This composite score was evaluated to determine where each child performed from mild autism to severe autism. Three groups were identified: severe (*n* = 43), moderate (*n* = 86), and mild (*n* = 49). Language development was determined by total score on the Preschool Language Scale 5th Edition.

## 3. Results

### 3.1. Prevalence

At the item level, results from the ATSC indicate high and widespread prevalence of abnormal tactile and other sensory responses as well as self-regulatory delay in children with autism. As can be seen in Table 2, oral-tactile prevalence is significantly higher than that for typically developing children (on a magnitude of two to four times) for the face and mouth areas. Prevalence patterns vary across the areas of the body, though they generally hold to the same pattern as with the face-mouth areas.

Consistent patterns of prevalence between the groups also emerge for several of the domains of self-regulatory delay. For example, in the areas of orientation-attending, self-soothing, sleep, and behavior, children with autism exhibit much higher prevalence of delay than do typically developing children. This pattern is not as strong in the areas of appetite-digestion and toileting.

Table 3 presents data on the percentage of children experiencing abnormal sensory responses and the areas of the body impacted by abnormal tactile response. These data represent the percent of children scoring at the mean plus one standard deviation of typically developing children in the study. Results are shown both for children with differing levels of severity of autism and for typically developing children. As can be seen here, children with differing levels of severity of autism experience higher levels of abnormal sensory response than do typically developing children, and children with severe autism experience higher levels of abnormal sensory response than do children with moderate or mild autism (except for smell). *χ* ^2^ analyses were conducted on the four sensory domains and the number of domains affected. All tests between the three levels of autism presented here and typically developing children were significant at the .0001 level. *χ* ^2^ values ranged from 187.9 for oral-tactile domain to 26.8 for smell (with 3 d.f.). Total number of senses affected was significant as well with *χ* ^2^ = 190.9 (d.f. = 12).

Parallel analyses were conducted on the areas of the body experiencing abnormal tactile response. Results were similar. All of the tests comparing children with differing levels of severity of autism to typically developing children were significant, with *χ* ^2^ values ranging from 171.6 for the mouth to 24.9 for the skin. The number of areas of the body affected was also significant with *χ* ^2^ value of 251.8 (d.f. = 21).

These results with a larger population and redesigned ATSC confirm previous reports that children with autism are distinguished by mixed pain and numbness of multiple areas of skin as well as global delay of early self-regulation milestones.

### 3.2. Reliability

Both internal consistency and test-retest estimates of reliability were calculated. We calculated internal consistency estimates (Cronbach's alpha) for the oral-tactile, orientation-attention, self-regulation, and other sensory domains and constipation and overall scores and a composite of face-mouth and orientation-attention. These results are shown in Table 4. Overall scale estimates were .873 for children with ASD and .899 for typically developing children. In the oral-tactile domain estimates of internal consistency were .708 for ASD and .791 for typically developing children. For the self-regulation domain, estimates were .769 for ASD and .794 for typically developing children. Subdomain scores demonstrated varying levels of internal consistency. The internal consistency of the composite for face-mouth and orientation-attention (a suggested set of items for screening purposes) is .764 for ASD and .763 for typically developing children.

Test-retest stability was estimated from a subsample of children in control group condition over a four-month time interval. Coefficients ranged from .763 for self-regulation to .806 for oral-tactile to .826 for the overall scale.

### 3.3. Discriminating between Populations

To test for the ability of the ATSC to discriminate between populations both MANOVA and ROC curve analyses were conducted. Table 5 contains the post hoc MANOVA results comparing children with autism and typically developing children. MANOVA results indicate that overall population means are significantly different across all domains and subdomains on the ATSC: overall, *F* = 123.3(6,397), *p* ≤ .000 with an effect size estimate of .651 (Partial eta^2^).

Post hoc analyses show that population means differ significantly across all domains and subdomains. Mean scores for children with autism are significantly different from mean scores for typically developing children.

ROC curve analysis was conducted to determine the sensitivity and specificity of the ATSC in accurately identifying children with autism. For the primary checklist of 47 items (oral-tactile, orientation-attention plus self-regulation) the area under the curve (AUC) is .961. A score of 49 produces sensitivity of 91.0 and specificity of 84.9. Oral-tactile scores (17 items) have an AUC of .935. A score of 17 produces sensitivity of 91.0 and specificity of 82.9. Orientation-attention scores (7 items) have an AUC of .933. A score of 7 produces sensitivity of 86.4 and specificity of 85.6. All of these are adequate to screen for autism, but require large numbers of items. Combining face-mouth and orientation-attention (12 items) produces an AUC of .953 and with a score of 13 produce sensitivity of .910 and specificity of .847. This combination of items powerfully and efficiently distinguishes between children with autism and typically developing children. The ATSC clearly distinguishes children with autism from typically developing children across all domains.

### 3.4. Discriminating between Levels of Autism

Parallel analyses were conducted comparing two levels of autism: severe and mild as determined by a composite of scores.  Table 6 contains the MANOVA post hoc results comparing children with different levels of autism. Overall MANOVA results indicate overall population means are significantly different between children with severe and mild autism, *F* = 17.32 (6,85), *p* < .000 with an effect size estimate of .550 (Partial eta^2^).

Post hoc analyses show only one domain (constipation) where specific population means do not differ significantly. Mean scores for severe and mild autism differ significantly on all other domains, with Fs ranging from 7.66 to 91.58.

ROC curve analysis was conducted to determine the sensitivity and specificity of the ATSC in accurately distinguishing between children with severe autism and those with mild autism. For the primary checklist of 47 items (oral-tactile, orientation-attention plus self-regulation) the area under the curve (AUC) is .918. A score of 76 produces sensitivity of 93.0 and specificity of 81.6. Both the total scale (59 items) and the composite of face-mouth and orientation-attention provide adequate sensitivity and specificity. Other domains do not. The overall scale has AUC of .908. A score of 90 produces sensitivity of 90.7 and specificity of 81.6. Combining face-mouth and orientation-attention (12 items) produces AUC of .884 and, with a score of 23, produces a sensitivity of 81.4 and specificity of 81.6. This combination of items provides an adequate and efficient screening tool to distinguish between children with autism and typically developing children. The ATSC is also able to distinguish between children with severe and mild autism.

## 4. Discussion

This study introduces clinicians to the Autism Touch and Self-Regulation Checklist and demonstrates that it is a reliable instrument with which to screen and manage tactile abnormalities in children with autism. The checklist assesses signs of peripheral tactile impairment by location and severity and has the additional benefit of confirming severity by measuring developmental impacts on early self-regulation. Existing sensory questionnaires are based on a sensory processing disorder model that does not distinguish between central and peripheral etiology of tactile symptoms [3, 4]. A central etiology for sensory problems cannot account for the high prevalence of tactile than other sensory abnormalities, the varying prevalence of tactile abnormalities on different areas of skin, or the findings of mixed pain and numbness reported in children with autism. In contrast, these are typical presenting signs of peripheral tactile impairment due to small fiber neuropathy [21, 22].

The first iteration of the ATSC explored whether symptoms were localized or generalized and mild or severe. Analysis indicated there was involvement of multiple areas of skin and the range of severity was more than twice that seen in typically developing children. Severity was confirmed by the finding of global delay of early touch-dependent, self-regulation milestones relative to orientation/attention, self-soothing, sleep, and digestion [6]. Thus, the evidence pointed to generalized tactile impairment that was severe enough to pose a barrier to parent/caregiver touch, resulting in self-regulatory delay. A subsequent outcome study showing improvement of self-regulatory delay following treatment of tactile abnormalities provided confirmation of a barrier to touch [11].

The hallmark of autism is social delay and it is argued that the search for the neurological basis of autism should focus on the social deficits [23]. An explanation for the early social deficits in autism has never been found. In our view, the search for the basis of social delay should focus on whether sensation is intact on the face. Social interaction in its earliest form is face-to-face interaction, and early damage to the sense of touch on the face would profoundly disrupt social development from the start. In Research Question #2 we screened the data pool for a direct relationship between evidence of damage to the sense of touch on the face and mouth and presence of social delay, that is, diagnosis of autism.

It was remarkable that five questions about touch/pain responses on the face and mouth should correctly identify 83% of the children with autism from typically developing children, especially since the questions bore little resemblance to the usual social and behavioral questions used to screen for autism. But intact oral-facial sensation is required for the orienting reflex to function. When we widened the net to include questions about failure to orient, 91% of the children with autism were correctly identified. This was an extraordinarily high yield, well above the usual yields from autism screening by parent questionnaire [24].

Oral-facial tactile impairment explains many things about social delay, especially the lack of attention to face seen in young children with autism [25]. Our results suggest that if the developmental hallmark of autism is social delay, then the physical hallmark of autism is tactile impairment of the face and mouth. And the connecting link between the two is failure of the orienting reflex to support early orientation to face and face-to-face attention.

One of the mysteries of autism is the spectrum of severity that it presents, as well as the basis on which the spectrum occurs. In using a tool designed to evaluate signs of tactile impairment, we hoped to identify a physical basis for that spectrum of severity. Our data described a spectrum of severity of tactile abnormalities ranging from mild withdrawal responses from texture of food in the mouth and trimming fingernails to mixed signs of pain and numbness on multiple areas of skin. In Research Question #3, we explored whether tactile impairment could provide physical basis for autism severity by seeking to differentiate severe from mild ASD on the basis of severity of tactile signs.

Over 80% of children with severe ASD were correctly identified from a pool of children with severe and mild ASD by severity of tactile scores. Given that the distinction is usually made on verbal and cognitive differences [26], it was perhaps astonishing that the two ends of the spectrum could be neatly identified without asking a single question about verbal or cognitive ability. But when the results were interpreted in light of what is known about early sensory impairment and development, they were similar to what would be expected from a range of hearing or vision impairment and exactly what would be expected from a range of tactile impairment. The conclusion is supported by treatment outcome research showing that reduction of severity of tactile abnormalities reduces severity of autism [12].

## 5. Conclusion

This paper presents data validating the reliability and usefulness of the Autism Touch and Self-Regulation Checklist to evaluate and manage tactile impairment in children with autism. The utility of identifying severity and location of tactile abnormalities is confirmed by finding that severity of tactile abnormalities was powerfully related to severity of autism, and location of impairment on the face and mouth efficiently screened the children with autism from the data pool. The finding that 91% of the children with autism could be correctly identified by signs of oral-facial tactile impairment and failure to orient illuminates the importance of the failure of the orienting reflex for the emergence of social delay in autism. Altogether, the findings add to mounting evidence of the importance of evaluating and managing tactile impairment at the time of autism diagnosis.

## Figures and Tables

**Table 1:** Validation group demographics.

	ASD (*N* = 199)	Typically developing children (*N* = 205)
Gender		
Male	170	103
Female	29	102
Age		
Mean	3.83 years	3.80 years
Standard deviation	1.10	.98
Range	2–6 years	2–6 years

Note: *N* = number of children.

**Table 2:** Item description and prevalence in children with autism.

Item	Autism spectrum disorder	Typically developing children
Oral-tactile domain		
Face-mouth		
Avoids foods with certain textures	78.9%	41.5%
Face washing is difficult	73.8%	20.0%
Tooth brushing is difficult	68.8%	18.0%
Eats very few foods	63.3%	16.6%
Mouths or chews objects	63.3%	18.1%
Head-hands-feet-skin-diaper		
Haircuts are difficult	72.3%	21.4%
Does not notice if the diaper is wet or dirty	60.3%	12.7%
Avoids wearing gloves	61.8%	16.6%
Cutting toenails is difficult	62.3%	24.4%
Does not cry tears when hurt	64.3%	14.6%
Refuses to wear a hat	56.2%	22.9%
Cutting fingernails is difficult	55.8%	23.9%
Head-bangs on soft surface	38.2%	2.0%
Will only wear certain footwear	36.7%	20.5%
Will only wear certain clothes	43.2%	24.4%
Cries tears when he or she falls, scrapes skin, or gets hurt	45.7%	56.6%
Head-bangs on a hard surface	28.2%	1.0%
Prefers to wear the same clothes day after day	29.7%	13.1%
Orienting-attending		
Stares off into space	73.9%	31.3%
Seems not to notice when spoken to in a normal voice	79.4%	18.0%
Does not respond to his or her name	72.8%	9.8%
Has to be prompted to make eye contact when spoken to	67.9%	25.4%
Seems unaware when others are hurt	73.4%	9.8%
Does not roll over onto back when asked	52.2%	1.0%
Does not notice or react when tapped on the back	43.2%	3.0%
Self-regulation domain		
Awakens very early and stays awake	65.8%	25.4%
Has difficulty falling asleep at bedtime	62.3%	33.7%
Has difficulty falling back asleep when he or she awakens during the night	64.4%	15.1%
Has difficulty awakening in morning	31.7%	14.6%
Will only eat familiar foods	83.9%	52.7%
Bowels are loose	51.2%	20.0%
Does not seem to be interested in food	50.7%	17.1%
Has difficulty calming when upset	87.4%	40.9%
Tantrums or meltdowns	80.4%	51.2%
Gets upset or tantrums when asked to make a transition	82.4%	29.8%
Cries easily when frustrated	71.9%	35.1%
Diaper is wet in the morning	69.9%	33.7%
Wears a diaper during the day	62.3%	13.2%
Is dry at night	52.5%	78.1%
Is toilet trained	46.2%	91.7%
Hits or kicks others	58.7%	35.1%
Throws things at others	18.1%	19.6%
Scratches or pulls others' hair	37.2%	10.3%
Bites others	29.2%	3.9%
Gets aggressive or hyper with exposure to certain smells	15.1%	1.0%
Hits self	28.2%	2.9%
Bites self	21.6%	0.0%
Pulls own hair	18.1%	1.5%
Other sensory domains		
Reacts strongly when others cry loudly or scream	59.8%	20.5%
Is startled by sudden noises	59.8%	34.2%
Covers ears with certain sounds	61.8%	32.2%
Reacts poorly to certain everyday noises	44.85	6.4%
Looks at objects out of sides of eyes	51.7%	24.4%
Is bothered by certain lights	48.85	13.2%
Gags with certain smells	27.2%	7.8%
Constipation		
Bowel movement is hard and dry	32.6%	14.2%
Bowel movements are often green	20.6%	3.9%
Bowel movements are twice a week	18.5%	9.3%
Bowel movement is once a week	14.0%	5.4%
Requires regular use of laxative to avoid constipation	13.5%	4.0%

**(a):** 

Group	Sensory domain impaired, %			
	Oral/tactile	Visual	Auditory	Smell
Typically developing children	17.6%	17.6%	17.1%	7.8%
Severe autism	100%	67.4%	72.1%	23.3%
Moderate autism	88.4%	66.3%	61.6%	25.6%
Mild autism	79.6%	38.8%	63.3%	18.4%

**(b):** 

Group	Number of senses impaired				
	4	3	2	1	0
Typically developing children	3.4%	5.9%	17.1%	29.8%	43.9%
Severe autism	37.2%	39.5%	14.0%	9.3%	0%
Moderate autism	30.2%	31.4%	24.4%	10.5%	3.5%
Mild autism	6.1%	36.7%	32.7%	20.4%	4.1%

**(c):** 

Group	Areas of the body affected						
	Face	Mouth	Head	Hands	Feet	Skin	Diaper
Typically developing children	20.0%	16.1%	13.2%	21.0%	21.0%	18.0%	12.7%
Severe autism	83.7%	95.3%	95.3%	79.1%	74.4%	37.2%	79.1%
Moderate autism	77.9%	81.4%	75.6%	70.9%	53.5%	45.3%	62.8%
Mild autism	67.3%	71.4%	61.2%	61.2%	59.2%	30.6%	40.8%

**(d):** 

Group	Number of areas of the body affected							
	7	6	5	4	3	2	1	0
Typically developing children	0%	1.0%	4.4%	3.9%	10.7%	10.2%	25.9%	43.9%
Severe autism	14.0%	41.9%	30.2%	7.0%	4.7%	0%	2.3%	0%
Moderate autism	9.3%	25.6%	26.7%	15.1%	10.5%	10.5%	2.3%	0%
Mild autism	0%	20.4%	26.5%	10.2%	24.5%	8.2%	6.1%	4.1%

**Table 4:** Internal consistencies of domains by group.

Domain	Cronbach's alpha		Number of items
	Autism total	Typically developing children	
Oral-tactile	.708	.791	17
Face-mouth	.625	.592	5
Head-hands-feet-skin-diaper	.607	.569	12
Orientation and attention	.760	.719	7
Self-regulation domains (sleep, appetite/digestion, self-soothing, toileting, and behavior)	.769	.794	23
Total oral-tactile, orientation and attention, and self-regulation	.864	.886	47
Other sensory domains (vision, hearing, and smell)	.694	.740	7
Constipation	.707	.599	5
Total scale	.873	.899	59
Face-mouth + orientation-attention	.764	.793	12

**Table 5:** Post hoc population comparisons.

	Autism *N* = 199	Typically developing children *N* = 205	*F* (d.f.)	*p* value
Oral-tactile				
M	27.60	10.66	525.1	<.000
SD	7.91	6.93	(1,402)	
Face-mouth				
M	10.06	3.74	412.1	<.000
SD	3.43	2.81	(1,402)	
Hands-feet-head-skin-diaper				
M	17.54	6.92	368.6	<.000
SD	6.08	5.00	(1,402)	
Orientation and attention				
M	12.58	3.99	522.6	<.000
SD	4.40	3.05	(1,402)	
Self-regulation total				
M	35.72	17.28	439.0	<.000
SD	9.75	7.86	(1,402)	
Oral-tactile, orientation-attention, and self-regulation				
M	75.90	31.93	685.1	<.000
SD	18.47	15.18	(1,402)	
Other sensory domains				
M	10.41	4.89	193.7	<.000
SD	4.32	3.63	(1,402)	
Constipation				
M	3.23	1.60	36.6	<.000
SD	3.21	2.13	(1,402)	
Total scale				
M	89.54	38.41	677.7	<.000
SD	21.35	18.03	(1,402)	
Face-mouth + orientation-attention				
M	22.64	7.73	663.0	<.000
SD	6.50	5.07	(1,402)	

Note: M = mean; SD = standard deviation; d.f. = degrees of freedom.

**Table 6:** Post hoc levels of autism comparisons.

	Severe autism *n* = 43	Mild autism *n* = 49	*F* (d.f.)	*p* value
Oral-tactile				
M	32.49	24.02	42.72	<.000
SD	5.19	6.97	(1,90)	
Face-mouth				
M	11.70	9.47	11.26	.001
SD	2.51	3.63	(1,90)	
Hands-feet-head-skin-diaper				
M	20.79	14.55	39.05	<.000
SD	4.03	5.35	(1,90)	
Orientation and attention				
M	15.47	9.94	55.24	<.000
SD	2.92	4.03	(1,90)	
Self-regulation total				
M	41.84	29.24	52.05	<.000
SD	7.14	9.29	(1,90)	
Oral-tactile, orientation-attention, and self-regulation				
M	89.87	63.20	91.58	<.000
SD	10.49	15.34	(1,90)	
Other sensory domains				
M	11.63	9.20	7.66	.007
SD	4.59	3.81	(1,90)	
Constipation				
M	2.84	2.57	0.25	.662
SD	2.62	2.53	(1,90)	
Total scale				
M	104.26	74.98	79.05	<.000
SD	13.55	17.46	(1,90)	
Face-mouth + orientation-attention				
M	27.16	19.41	58.86	<.000
SD	4.03	5.45	(1,90)	

Note: M = mean; SD = standard deviation; d.f. = degrees of freedom.
